# Observation of Ultrashort Laser Pulse Evolution in a Silicon Photonic Crystal Waveguide

**DOI:** 10.3390/mi12080911

**Published:** 2021-07-30

**Authors:** Xiaochun Wang, Jiali Liao, Jinghan Pan, Heng Yang, Xiujian Li

**Affiliations:** 1 Department of Physics, College of Liberal Arts and Sciences, National University of Defense Technology, Changsha 410073, China; xiaochunwang01@163.com (X.W.); panjinghan18@nudt.edu.cn (J.P.); 2 School of Physics and Optoelectronic Engineering, Xidian University, Xi’an 710071, China; liaojiali@xidian.edu.cn; 3 College of Information and Communication, National University of Defense Technology, Changsha 410073, China; hengy@yeah.net

**Keywords:** photonic crystal waveguide, pulse acceleration, blue shift

## Abstract

Using the sum frequency generation cross-correlation frequency-resolved optical gating (SFG-XFROG) measurement setup, we observed the soliton evolution of low energy pulse in an Si photonic crystal waveguide, and it exhibited the pulse broadening, blue shift, and evident pulse acceleration. The soliton evolution was also investigated by nonlinear Schrödinger equation (NLSE) modelling simulation, and the simulated results agreed well with the experimental measurements. The effects of waveguide length on the pulse evolution were analyzed; the results showed that the pulse width changed periodically with increasing waveguide length. The results further the understanding of the ultra-fast nonlinear dynamics of solitons in silicon waveguides, and are helpful to soliton-based functional elements on CMOS-compatible platforms.

## 1. Introduction

With the capability to tightly confine optical modes [1], the compatibility with mature CMOS processes [2], and the extremely large nonlinearities enhanced by slow-light effects, the Si photonic crystal waveguides (PhCWs) have been attracting more and more intention. Four-wave-mixing, temporal soliton, optical filter, all-optical modulation, all-optical switching, ultralow-power all-optical signal processing and all-optical wavelength conversion have been demonstrated in Si PhCWs [1,3]. Basically, pulse propagation is affected by the nonlinear properties and the inherent dispersion of the Si PhCWs, and they can make the pulse propagation exhibit optical solitons effects at the anomalous dispersion region [4], which will be important for numerous applications of optical information processing [5,6,7,8]. Recently, the linear and nonlinear effects such as the group velocity dispersion (GVD), the self-phase modulation (SPM), two photon absorption (TPA), cross phase modulation (XPM), have been widely studied [9,10,11,12,13]. As the solitons are the nonlinear waves that exhibit invariant or recurrent behavior as they propagate through some materials [14,15], measuring and governing its propagation by the precise control of the dispersion and nonlinear effects is very helpful for many applications [16,17].

Actually, the FROG and SFG-XFROG are efficient tools for check the measurements of ultra-short laser pulses [18,19,20]. However, up to now, although we had checked the soliton behavior in the Si nanowire and PhCWs [21,22,23,24,25,26], there is unclarity about all details of the soliton propagation, especially the evolution dynamics of the low-energy soliton in the Si PhCWs, which are considered to be suitable to manage the solitons for the Si nanowire [27].

Herein, based on a sensitive an SFG-XFROG ultra-short laser pulse measurement setup, we observed the picosecond pulse evolution, which shows the pulses blue shift and pulse shaping in Si PhCWs with low input pulse energy of several pico-joule. The pulse evolution has also been investigated by the optimized nonlinear Schrödinger equation (NLSE) modeling simulation, and the NLSE predicted results agree well the experimental measurements. The research results provide valuable information for the design of waveguides [28] for various on-chip photonic circuits, in support of the ultra-broadband high-speed optical communications and optical signal processing.

## 2. Measurement Setup and Observations of Pulse Evolution

**Figure 1:**
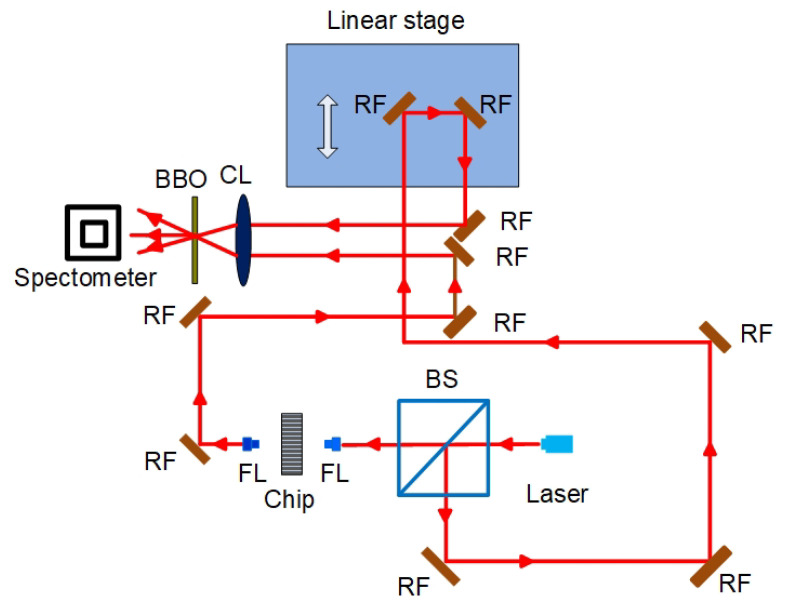
Experimental measurement setup of SFG-XFROG (RF: Reflector, BS: Beam splitter, FL: Focusing lens, CL: Bi-convex lens, BBO: Barium borate crystal).

The SFG-XFROG experimental measurement setup for the ultrashort pulse characterization is shown in Figure 1, and it can provide sensitive and accurate measurement of the optical pulses output from the silicon photonic crystal waveguides in the time and spectral domains. A BBO crystal (1 mm thickness, provided by CASTECH INC. CHINA) and a Horiba JY FHR1000 spectrometer with a SYMPHONY II UVCCD-1024 × 256-BIDD detector were applied to enable the minimum detected pulse energy to be as low as 500 attojoule (aJ), the spectral resolution to be better than 0.1 nm, and the time resolution to be less than 1 femtosecond (fs). The measurement setup provides the detailed properties of the ultrashort pulses, including the time and frequency intensity and phase distributions, which help to capture the soliton dynamics. The Si PhCWs used in this work are the same as Ref. [26].

**Figure 2:**
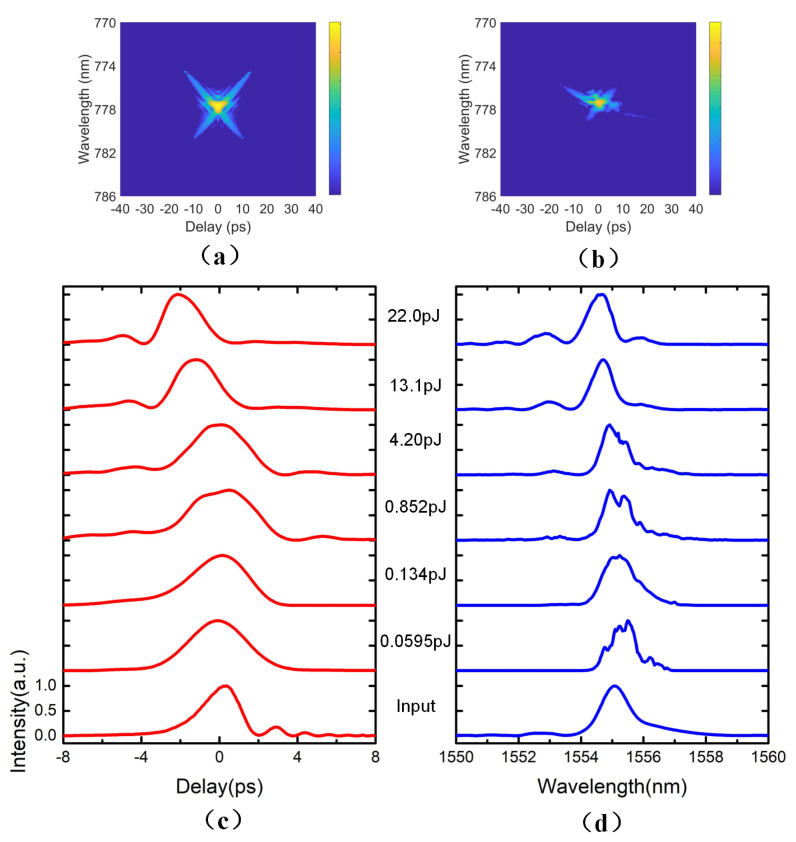
The output SFG-FROG/XFROG measurements, (**a**) FROG trace of the input pulse, (**b**) XFROG trace of the output pulse corresponding to Ep = 22.0 pJ, (**c**) the normalized intensity profiles in temporal domain, (**d**) the normalized intensity profiles in the spectral domain.

Utilizing the SFG-FROG, we have measured the input pulse, and the measured trace of the input pulse is shown in Figure 2a; the temporal and spectral intensity profiles are extracted from this trace, and shown in the bottom of Figure 2c,d. The FROG retrieving error is as low as 0.075% for the reconstruction processes. The pulse duration and central wavelength of the input pulse are 2.25 ps and 1555 nm, respectively. The output pulses from the Si PhCWs with increasing input pulse energies $E_{p}$ have been characterized using the SFG-XFROG, and the measured trace at $E_{p}=22$ pJ is shown in Figure 2b, where the XFROG retrieving error is 0.13%.

The experimental results demonstrate that the output pulse is broadened after passing the waveguide as shown in Figure 2c. The broadening effect of the output pulse weakens as the FWHM of the output pulse shrinks from 3.91 ps to 2.50 ps, corresponding to a broadening factor ($FWHM_{output}/FWHM_{input}$) from 1.74 to 1.11, with the pulse energy increasing from 59.5 fJ to 13.1 pJ. However, at the pulse energy of 22.0 pJ, the pulse exhibited slight compression with the compression factor ($FWHM_{output}/FWHM_{input}$) of 0.98. The temporal domain profile shows that the FWHM of the output pulse is affected by the input pulse energy. The FWHM of the output pulse was broadening first and then compressing with the increasing of input pulse energy. The output pulse shows obvious pulse acceleration. The largest pulse acceleration is 2.17 ps with 22.0 pJ input energy. The output pulse is still smooth and not destroyed in the experiments.

The Figure 2d shows the normalized intensity profiles of the output pulses in spectral domain. The pulse shows red shift with the low input pulse energy and blue shift with the high input pulse energy in Figure 2d. The largest red shift is 0.41 nm with an input energy of 59.5 fJ, and the largest blue shift is 0.68 nm with an input energy of 22.0 pJ.

## 3. Optimized NLSE Modeling Simulations and Discussions

In order to unveil the principles of the pulse broadening and spectrum shift as shown in Figure 2, we perform the optimized nonlinear Schrödinger equation (NLSE) modeling simulations [26].

(1) $$\frac{\partial A}{\partial z}+i\frac{\beta_{2}}{2}\frac{\partial^{2}A}{\partial^{2}t}-\frac{\alpha_{eff}}{2}A=i\left(\gamma_{eff}-\alpha_{TPA.eff}\right){\left|A\right|}^{2}A+\left(ik_{0}k_{c.eff}-\frac{\sigma_{eff}}{2}\right)N_{c}A$$

(2) $$\frac{\partial N_{c}\left(z,t\right)}{\partial t}=\frac{\alpha_{TPA.eff}}{2hv_{0}}{\left|A\left(z,t\right)\right|}^{4}-\frac{N_{c}\left(z,t\right)}{\tau_{c}}$$

where the group velocity dispersion (GVD) $\beta_{2}=-1800$ ps${}^{2}$/m; The Kerr coefficient is $1.02\times 10^{3}$ (1⁄W/m); The effective TPA coefficients is $13.4\times 10^{-12}$ m/W; The effective free carrier dispersion and absorption parameters are $k_{c.eff}=-13.3\times 10^{-27}$ m${}^{3}$ and $\sigma_{eff}=4.62\times 10^{-21}$ m${}^{2}$, respectively. *A* is the slow varying envelope of the propagation pulse electric amplitude; $N_{c}$ is the density of free carrier; *Z* is the pulse transmission position inside the waveguide. $\tau_{c}$ is the free carrier effective lifetime, which is estimated to $0.5\times 10^{-9}$ s. *h* and $v_{0}$ are the Plank constant and light frequency, respectively [26].

**Figure 3:**
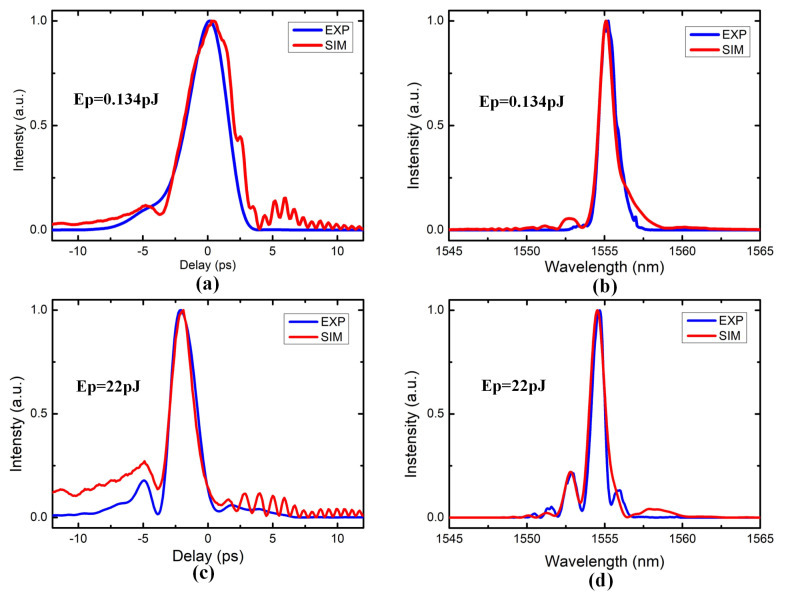
The experimental and the optimized NLSE simulation results for various pulse energies, (**a**,**c**) temporal profiles, (**b**,**d**) spectral profiles of the pulse, (**a**,**b**) with 0.134 pJ input pulse energy, (**c**,**d**) with 22.0 pJ input pulse energy. The blue solid line are experimental results, and the red solid line are optimized NLSE simulation results.

Figure 3 shows the experimental and simulation results for 1555 nm with the input pulse energy of 0.134 pJ and 22.0 pJ, respectively. In the simulations, the input pulse profile measured by SFG-FROG is used as the initial pulse. Obviously, the major experimental results are matched with the NLSE simulation results, which indicates that the optimized NLSE modeling and the parameters are suitable for the Si PhCWs, which illustrate the NLSE modeling simulation can properly predict pulse evolution dynamics in the Si PhCWs. However, we can also find that, some slight periodic oscillation side wings appear around the main peak of the simulation results, which are induced by the FFT-window selection of the split-step solution method for the NLSE, and can be considered noise perturbations of the simulation.

**Figure 4:**
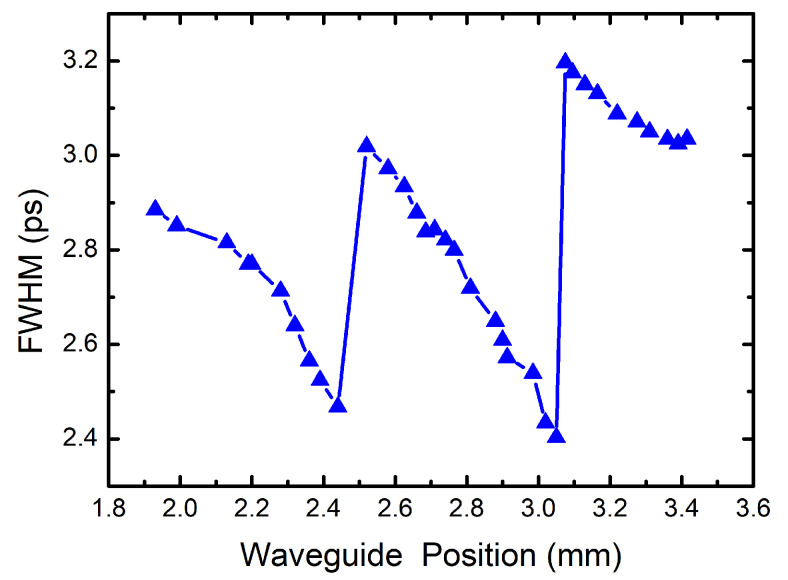
The FWHM of output pulse temporal profiles by the NLSE simulations with different waveguide position for 22.0 pJ input pulse energy at 1555 nm.

The input pulse for the simulations is the experiment input pulse at 22.0 pJ with center wavelength 1555 nm. By increasing the Si PhCW length, we calculate the output pulse widths at different propagation distances, and the results are shown in Figure 4. It is shown that the pulse temporal FWHM is transformed periodically with increasing propagation distance. In each period, the temporal FWHM is gradually compressed to the narrowest, and then sudden broadening occurs in the transmission following the maximum compression. Because of the large losses in the Si PhCWs, the minimum pulse width increases with the soliton evolution periods. The pulse transmission emerges the soliton with the balance between the nonlinear effects and the dispersion in the Si PhCWs.

## 4. Conclusions

In summary, using sensitive SFG-XFROG measurements, we observed pulse broadening with a broaden factor of 1.74, blue shift, red shift and obvious pulse acceleration when the temporal soliton transmitted through the Si PhCW at a center wavelength of 1555 nm. The optimized NLSE simulation results matched the measurements. The results show that the FWHM of pulse gradually changes to the narrowest, and then broadening occurs following the narrowest point along the propagation distance, i.e., the simulation results show that periodic solitons are produced in the process of pulse transmission. Through the study, we can optimize the Si PhCWs for pulse shaping. These results help us understand how ultra-fast pulses propagate in silicon-based waveguides, and even open the way for soliton-based functional elements in CMOS-compatible platforms.
